# Plant macrofossil evidence for an early onset of the Holocene summer thermal maximum in northernmost Europe

**DOI:** 10.1038/ncomms7809

**Published:** 2015-04-10

**Authors:** M. Väliranta, J. S. Salonen, M. Heikkilä, L. Amon, K. Helmens, A. Klimaschewski, P. Kuhry, S. Kultti, A. Poska, S. Shala, S. Veski, H. H. Birks

**Affiliations:** 1 Department of Environmental Sciences, ECRU, University of Helsinki, P.O. Box 65, Helsinki FI-00014, Finland; 2 Department of Geosciences and Geography, University of Helsinki, P.O. Box 65, Helsinki FI-00014, Finland; 3 Institute of Geology, Tallinn University of Technology, Ehitajate tee 5, Tallinn 19086, Estonia; 4 Department of Physical Geography, Stockholm University, Stockholm 106 91, Sweden; 5 School of Geography, Archaeology and Palaeoecology, Queen's University, Belfast, Northern Ireland BT7 1NN, UK; 6 Department of Physical Geography and Ecosystem Analysis, Lund University, Sölvegatan 12, Lund 223 62, Sweden; 7 Department of Biology and Bjerknes Centre for Climate Research, University of Bergen, P.O. Box 7803, Bergen N-5020, Norway

## Abstract

Holocene summer temperature reconstructions from northern Europe based on sedimentary pollen records suggest an onset of peak summer warmth around 9,000 years ago. However, pollen-based temperature reconstructions are largely driven by changes in the proportions of tree taxa, and thus the early-Holocene warming signal may be delayed due to the geographical disequilibrium between climate and tree populations. Here we show that quantitative summer-temperature estimates in northern Europe based on macrofossils of aquatic plants are in many cases *ca*. 2 °C warmer in the early Holocene (11,700–7,500 years ago) than reconstructions based on pollen data. When the lag in potential tree establishment becomes imperceptible in the mid-Holocene (7,500 years ago), the reconstructed temperatures converge at all study sites. We demonstrate that aquatic plant macrofossil records can provide additional and informative insights into early-Holocene temperature evolution in northernmost Europe and suggest further validation of early post-glacial climate development based on multi-proxy data syntheses.

## Abstract (web summary)

A geographical disequilibrium between climate and tree populations may bias northern European pollen-based temperature reconstructions for the early Holocene. Here, the authors compare pollen- and macrofossil-based temperature reconstructions, showing that macrofossils reveal warmer July temperatures.

## Graphical abstract

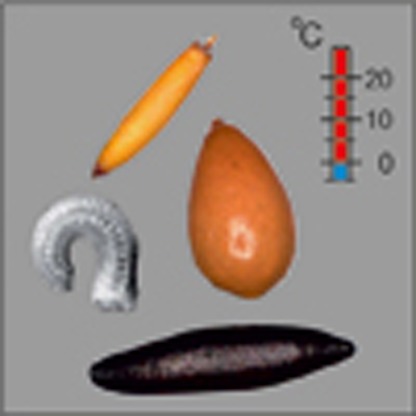

Glacial climate warmed irregularly during the last glacial termination (late glacial, *ca*. 14,700–11,700 cal BP (BP=before 1950 AD)) and then warmed rapidly to interglacial values after the start of the Holocene. During the first Holocene millennia, summer temperatures were up to 2 °C higher than today (Holocene Thermal Maximum). This was most strongly marked at high northern latitudes due to orbital variations that resulted in high summer insolation. After the main ice sheet had melted and the North Atlantic circulation had stabilized in interglacial mode, summer temperatures exceeded those of today, peaking between *ca*. 9,000 and 7,500 cal year BP^1^.

Most early Holocene temperature reconstructions are derived from fossil terrestrial–pollen assemblages that exclude local elements such as aquatic plants and assume that the pollen record reflects the regional vegetation and therefore climate changes^2^,^3^. They do not take into account the delay in tree range expansion in response to early Holocene climate warming due to the long population doubling times and relatively slow spread of trees^4^.

In his classic work on the late-glacial flora of Denmark, Iversen^5^ proposed that aquatic plants may provide a more accurate temporal record of the late-glacial and early post-glacial climate changes than trees due to their more rapid dispersal rates. However, studies on the post-glacial dispersal history of aquatic plants are very rare. Pollen records show that aquatic plants closely followed the melting ice-margin and reached their modern ranges in North America during the early Holocene^6^,^7^. As quantitative climate reconstructions using aquatic-macrophyte data are scarce (see refs 8, 9), the climate-indicator value and broad-scale dispersal dynamics of aquatic plants in the Holocene are still poorly understood^10^,^11^. Modern studies suggest that bird transport and spreading along water systems^11^ are the main and fastest means of dispersal. Early Holocene macrofossil records from lake sediments are often characterized by relatively diverse aquatic plant assemblages^8^,^12^,^13^ implying rapid early-Holocene colonization during a period when terrestrial pollen assemblages may reflect vegetation that is out of equilibrium with climate and with low diversity, and possibly low productivity^14^.

Holocene pollen-based summer temperature reconstructions from northern Europe are predominantly driven by changes in the major arboreal pollen taxa^2^, and inferred rises in temperature typically coincide with increases in certain tree-pollen taxa, such as spruce (*Picea abies*) in north-eastern European Russia^15^, pine (*Pinus sylvestris*) in northern Fennoscandia^16^ and deciduous trees (*Alnus incana*, *Alnus glutinosa*, *Ulmus glabra*, *Corylus avellana*, *Tilia cordata* and *Quercus robur*) in southern Fennoscandia and the Baltic countries^17^ (Supplementary Fig. 1a). We hypothesize that in such tree-pollen-driven temperature reconstructions, the early-Holocene climate warming signal may be delayed as a result of disequilibrium between climate and tree species distribution and population dynamics^14^,^18^. This possible shortcoming of pollen-based climate reconstructions needs to be examined by independent data, especially at glacial–interglacial transitions, because pollen data are the most commonly used proxy data source in palaeo data–climate modelling intercomparison exercises^1^,^19^. Suitable independent data to test this hypothesis can be provided by macrofossil records of aquatic plants. Owing to their more rapid response times, aquatic plants may reflect actual temperature changes faster than pollen-based temperature reconstructions.

Pollen-based temperature reconstructions are generally based on pollen–climate calibrations prepared using large data sets of modern pollen (sediment core-top assemblages) and corresponding climate variables (measured climate normals)^3^,^20^. Instead of proportional down-core data and surface-sediment calibration sets, macrofossil reconstructions can use information about the presence of individual indicator taxa in macrofossil assemblages and their modern distribution ranges and ecological requirements, as demonstrated in Väliranta *et al*.^21^ for an early-Weichselian interstadial sequence from southern Finnish Lapland (see also refs 5, 22, 23, 24, 25, 26).

In this study we compile and compare July temperature (*T*_jul_) reconstructions based on aquatic and telmatic (helophytes) plant macrofossils (henceforth *T*_julM_), and terrestrial pollen (henceforth *T*_julP_). The results support our hypothesis that temperature reconstructions based on macrofossils of aquatic taxa that can spread and respond faster than long-lived trees, detect early-Holocene temperature rises before pollen-based reconstructions in the study area. The latter are strongly dominated by the rises of pollen of the major tree-pollen taxa, which were still spreading towards their climatic limits and were therefore out of equilibrium with summer temperatures.

## Results

### Study location

**Figure 1:**
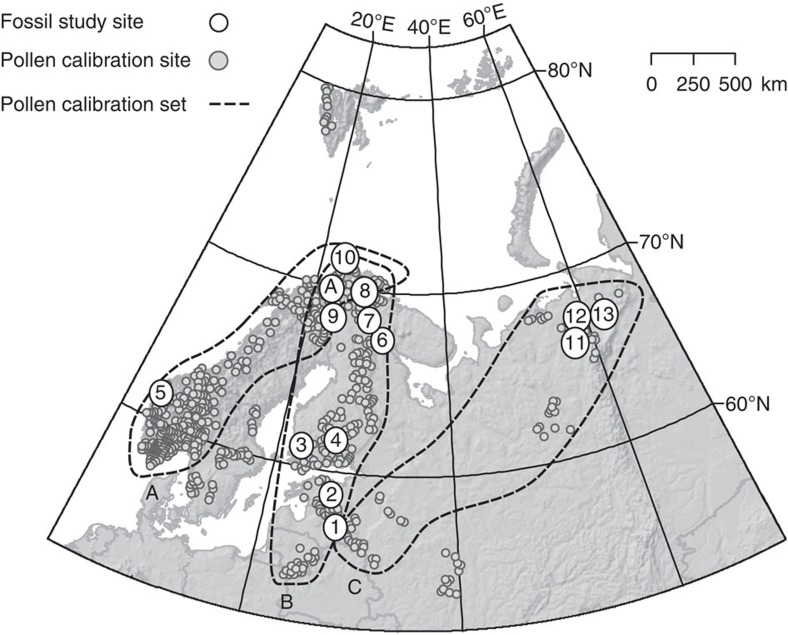
Locations of the study sites. The 13 study sites selected represent four different biomes: boreonemoral, southern boreal, northern boreal and subarctic-arctic vegetation zones. Fossil pollen and macrofossil study sites are indicated with numbered circles. (1) Lielais Svetinu, (2) Nakri, (3) Kankaanjärvi, (4) Laihalampi, (5) Kråkenes Lake, (6) Loitsana, (7) Kipojärvi, (8) Njargajavri, (9) Korsajavri, (10) Jansvatnet, (11) Llet-Ti, (12) Tumbulovaty and (13) Kharinei. See Supplementary Table 1 for details and references about these sites. The study site locations with pollen data adopted from the literature, Lake Tsuolbma^16^ and Toskal^67^, are indicated by capital letter A. The pollen–climate calibration data sets were selected from the 583-sample surface-pollen data set of Salonen *et al*.^59^, marked with small circles. To represent the modern pollen–climate relationships in the continentality regimes of different fossil sites, three different subsets (A: low continentality, B: intermediate continentality, C: high continentality) were selected from all the surface pollen assemblages. The modern *T*_jul_ gradient varies from 17.7 °C (Latvia) to 7.5 °C (northern Fennoscandia) and the annual precipitation varies between 1,280 mm (west coast of Norway) and 500 mm (Finnish Lapland). The elevation range of the sites is 38–705 m.a.s.l.

Finland is relatively flat with low mountains only in the northwest. The oceanity–continentality gradient is not particularly marked today: in general, continentality increases from the southwest (semi-oceanic) to the northeast (semi-continental). Likewise, the precipitation gradient is gradual, with a difference of *ca*. 200 mm a^−1^ between southern and northern Finland. The country stretches over 10° of latitude, resulting in a pronounced south–north temperature gradient. The *T*_jul_ ranges from *ca*. 17 °C in the south–southeast to *ca*. 7.5 °C in the mountains of western Lapland. As a result, Finland spans several bioclimatic zones; the boreo-nemoral (the southernmost bioclimatic zone characterized by mixed coniferous and broad-leaved forests), the boreal (dominated by coniferous forests) and the subarctic vegetation belts (between the boreal zone and the tree line which, in Finland, is formed by mountain birch). Therefore, many plant species reach their northern distribution limits within Finland. Only in the northernmost part of the country are plant distributions limited by elevation-related climatic conditions (orohemiarctic). Unlike many European countries where human activities strongly influence plant distributions, Finland can be considered to be in a relatively natural state. These geographical facts provide an excellent setting to exploit observed modern species–temperature relationships for palaeoecological temperature reconstructions. To facilitate discussion, the study sites (Fig. 1) are regionally clustered as follows: boreonemoral–southern boreal (Lakes Lielais, Nakri, Kankaanjärvi and Laihalampi), oceanic boreal (Kråkenes Lake), northern boreal (Lakes Loitsana and Kipojärvi) and subarctic (Lakes Njargajavri, Korsajavri and Jansvatnet). Their data are supplemented by literature records from lakes Tsuolbma and Toskal in northern Finland, and records from northeastern European Russia where the lakes are located in different bioclimate zones: northernmost boreal taiga (Lake Lleti), arctic tree line (Lake Tumbulovaty) and arctic tundra (Lake Kharinei). In the comparison of the pollen- and macrofossil-based reconstructions, the time frame 11,700–7,500 cal BP is divided into four time windows: 11,700–10,500, 10,500–9,500, 9,500–8,500 and 8,500–7,500 cal BP.

### Site-specific and regional *T*_jul_ reconstructions

**Figure 2:**
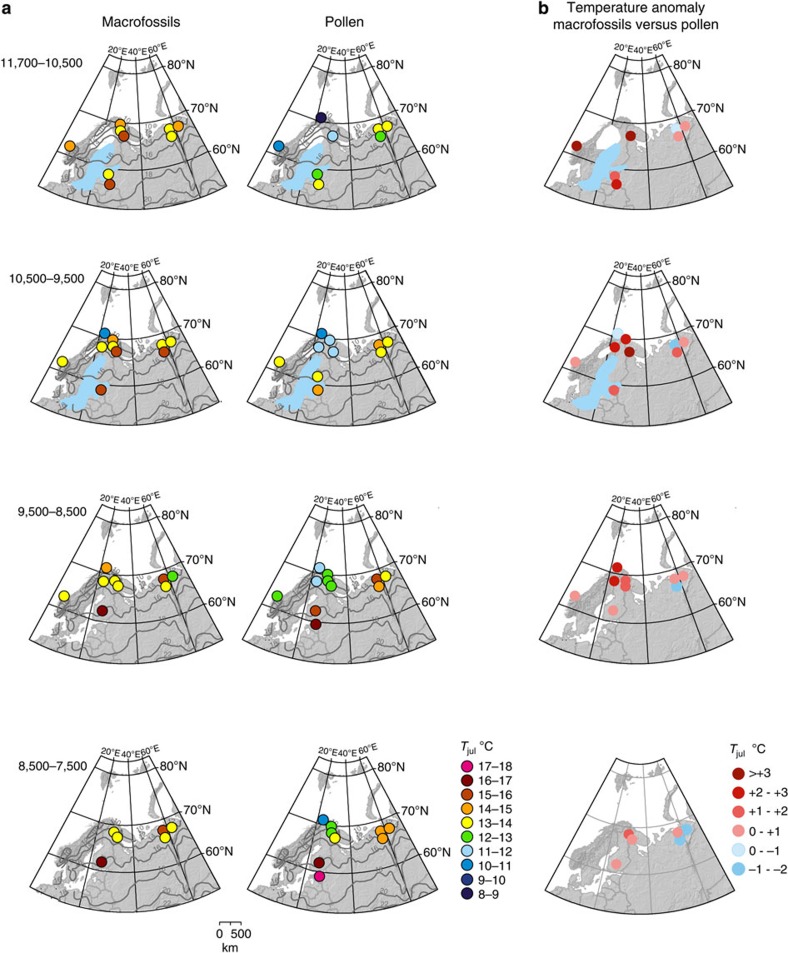
A comparison of macrofossil- and pollen-based mean *T*_jul_ in northern Europe for the four early post-glacial time windows. It should be noted that in Russia, the mean s.e. for pollen are somewhat higher at 1.05–1.15 °C, probably because the Russian calibration data set is smaller and the species-response estimates are thus less certain. (**a**) The spatio-temporal pattern of macrofossil- and pollen- based mean July temperatures (*T*_julP_ and *T*_julM_, respectively) in early- and mid-Holocene time windows (Supplementary Table 3). The pollen-based *T*_julP_ for each site is calculated as the median of all reconstructed values from fossil samples dating to the time window in question. The macrofossil-based *T*_julM_ is the highest site-specific value for the time window in question (see Methods for details). In addition, essential information about the ice-margin position and post-glacial stages of the Baltic Sea are shown (adapted and modified from ref. 38). Modern *T*_jul_ isolines (in grey) are also presented. (**b**) Temperature anomalies between *T*_julM_ and *T*_julP_ reconstructions (Supplementary Table 3). Red colour indicates positive anomalies where macrofossil-based temperatures are higher than pollen-based temperatures, while blue colour indicates negative anomalies. Note that pale pink and pale blue spots can both indicate no difference (0 °C). However, it should also be noted that due to the nature of *T*_julM_ as a lowest estimate for mean *T*_jul_, negative *T*_julM_–*T*_julP_ anomalies are difficult to interpret, as it is impossible to verify whether the anomaly is for instance due to a false absence of macrofossils. In contrast, positive *T*_julM_–*T*_julP_ anomalies can be more confidently taken to suggest a negative bias in the *T*_julP_ values (see Methods for more detailed methodological discussion and data interpretation). If pollen data were not available from the same lake as macrofossil data, the *T*_julM_ was compared with *T*_julP_ derived from an adjacent lake from the same climate zone.

The time-window values derived for *T*_julM_ and *T*_julP_ are shown in Fig. 2a. Full site-specific reconstructed *T*_jul_ curves are presented in Supplementary Fig. 2.

Before the time-window 8,500–7,500 cal BP, almost all *T*_julM_ reconstructions show warmer temperatures than *T*_julP_ in all study regions, although some sites, especially in Russia, occasionally show slightly negative anomalies (Fig. 2b). In Russia, the positive anomalies are also slightly smaller, 1–2 °C, than elsewhere for the earliest part of the Holocene. Overall, the largest positive *T*_jul_ anomalies occur in the north and the difference becomes smaller towards the mid-Holocene (Fig. 2 and Supplementary Fig. 2).

It should be mentioned that a few subarctic macrofossil records from Finland are fragmented as the sedimentation was discontinuous, probably due to fluctuations in water level. For example, Lake Njargajavri was filled by aquatic bryophytes at *ca*. 10,500 cal BP and sedimentation stopped for an unknown period of time^27^. Nevertheless, macrofossils suggest higher temperatures than pollen for the earliest intervals in such records, with *T*_julM_ in subarctic Finnish Lapland >13 °C since *ca*. 11,000 cal BP, when sedimentation started, while *T*_julP_ was around 10–11 °C or less (Fig. 2a).

Supplementary Fig. 3 illustrates the regional-scale early-Holocene temperature development through time windows. In all regions, *T*_julM_ are higher than *T*_julP_ for the earliest time windows. Moreover, towards mid-Holocene only in the boreo-nemoral and southern boreal regions do the *T*_julP_ temperatures exceed *T*_julM_, but it should be noted that for 9,500–8,500 and 8,500–7,500 cal BP time windows, macrofossil data are available only from Southern Finland.

## Discussion

Recently, post-glacial tree migration-rate estimates have been revised in the light of emerging macrofossil evidence for the persistence of small tree populations in central and northern Europe, the Baltic region and European Russia during the Last Glacial Maximum and the Late Glacial (14,500–11,700 cal BP)^28^,^29^,^30^,^31^,^32^,^33^. The previous fast migration rate estimates (100–1,000 m per year), calculated on tree population spread from southern European refugia^34^, have been reduced to 60–260 m per year^35^. Trees reached our study sites at different times during the early Holocene (Supplementary Fig. 1b). Pollen and macrofossil records from northeastern European Russia^30^ show that the response times of small northern tree populations to climatic change can be in the order of many centuries. However, faster response times have been reported from the Baltic region during the last glacial–interglacial transition and the 8.2-ka cold event^28^,^29^,^36^.

The glaciation histories of Fennoscandia, the Baltic region and European Russia differ considerably. Northern European Russia has been ice-free for the last *ca*. 90,000 years^37^. In the Baltic region, deglaciation and the following phases of the ancient Baltic Sea led to a slow emergence of land since *ca*. 15,000 cal BP^38^ (Fig. 2). Here, macrofossil evidence indicates that populations of tree birch (*Betula pendula*), pine (*P. sylvestris*) and aspen (*Populus tremula*) were present very close to the melting margin of the Fennoscandian Ice Sheet from 14,000 to 13,500 cal BP, while spruce (*P. abies*) immigrated at *ca*. 12,500 cal BP^28^,^29^,^39^. In addition, broad-leaved trees spread to the Baltic region in the early Holocene around 10,500–10,000 cal BP when the Fennoscandian Ice Sheet was reduced to a small area in northern Fennoscandia^17^,^40^. Hence, the influence of tree-population development and migration lags on the pollen-based reconstructions may be less pronounced in the Baltic region than further north.

In northeastern European Russia, *T*_julP_ and *T*_julM_ show more similar temperature development. Macrofossils infer the same or slightly higher regional *T*_julM_ than *T*_julP_ (<15 °C) (Fig. 2) for the earliest Holocene up to 7,500 cal BP. Independent *T*_jul_ reconstructions from chironomid data of >14 °C from one of our sites, the tundra Lake Kharinei^41^ (13 on Fig. 1) support the *T*_julM_ reconstructions at around 15 °C. In addition, macrofossil data at sites to the southwest in Karelia and the Yaroslavl' region also suggest early-Holocene *T*_jul_ up to 15 °C (refs 13, 42). We note that in Russia our macrofossil-derived *T*_julM_ of 15.7 °C is solely due to the presence of *Typha* seeds. At present, *Typha latifolia* and *Typha angustifolia* have very clear distribution ranges that seem to follow regional climate parameters (Supplementary Fig. 4, Den Virtuella floran; http://linnaeus.nrm.se/flora/welcome.html). The northern limit of continuous *T. latifolia* presence corresponds to *ca*. 17 °C in more continental European Russia (Den Virtuella floran), whereas our reconstructed temperatures are based on plant distributions in Finland. *Typha* is often one of the first taxa detected in macrofossil records^13^ (Supplementary Fig. 5). It is a very prolific, wind-dispersing, early successional taxon. It can rapidly colonize new areas, from one seed to 58 m^2^ cover in 2 years in suitable habitats^43^. Moreover, if the reported current distribution range of *Elatine hydropiper* in European Russia (Den Virtuella floran) is accurate, its presence at *ca*. 11,000 cal BP in the Lake Kharinei sequence would also indicate *T*_julM_ greater than 17 °C, supporting the climatic inferences provided by the presence of *Typha.* Therefore, if Russian distribution data are used, early Holocene *T*_julM_ was *ca*. 2 °C higher than *T*_julP_. This highlights a difficulty posed by obtaining reliable and detailed distributional data and their correlation with reliable meteorological data. As mentioned, temperature limits tend to become higher in more continental areas and regional variation should ideally be taken into account.

The most striking difference between the macrofossil- and pollen-based reconstructions is exhibited in subarctic and northern boreal Fennoscandia, with macrofossils inferring 2 °C–4 °C higher temperatures until 8,500 cal BP (Fig. 2). The reconstructions are based on relatively numerous aquatic and other indicative macrofossils. Several aquatic species suggest a *T*_julM_ of at least *ca*. 13–14 °C, but the presence of *Typha* and *Glyceria lithuanica* in the northern boreal sites indicates a *T*_julM_ >15 °C.

Macroscopic Scots pine (*P. sylvestris*) remains show that this species was present in the northern boreal zone from 9,500 cal BP onwards (Supplementary Fig. 1b). The delayed *T*_julP_ rise may reflect its late spread and low pollen productivity near its northern limit^44^. It never reached the highest and the northernmost sites discussed here. In addition, it is possible that the early-Holocene vegetation contained local taxa, such as sedges and grasses whose abundant pollen has a relatively poor temperature-indication value, thus depressing tree percentages and the pollen-based temperature signal. Northern Fennoscandia therefore contrasts with northeastern European Russia and the southern sites where *T*_jul_ was higher and boreal trees were already present during the late glacial. The slow northward spread of trees resulted in the greatest discrepancy between *T*_julP_ and *T*_julM_ in Northern Fennoscandia.

Aquatic plants have the ability to disperse rapidly within their climatic tolerances, and so after major climate warming they will rapidly colonize new suitable habitats. If the recent aggressive spread of the introduced *Elodea canadensis* in Finland of >50 km per decade^45^ is typical, aquatic plants could spread from the Last Glacial Maximum ice-margin in continental Europe to the coast of the Arctic Ocean in about 500 years. High early-Holocene insolation^46^ leading to warm summers combined with the weak summer temperature gradient between 60° and 70°N during the early Holocene^47^ enabled rapid northward spread of aquatic plants. New lake habitats were rapidly colonized through frequent and continuous dispersal and the lack of geographical barriers^11^. In North America^6^,^7^, aquatic species spread rapidly along the melting ice-sheet margin, reaching their modern distribution ranges early in the Holocene, implying effective reproduction in suitable ecological conditions including warm summer temperatures. This pattern was probably replicated in northern Europe. In contrast, *T*_julP_, largely dependent on tree pollen taxa, does not detect this early rapid warming. In contrast to aquatics, long-lived tree species have traits and requirements that lead to relatively slow population establishment with a population doubling time of 20–500 years^4^, low pollen production at distribution limits^44^ and soil requirements that were not met immediately after the ice-sheet melted^48^.

Holocene summer temperatures in the Northern Hemisphere follow the gradually decreasing orbitally forced summer insolation after 11,000 cal BP^46^. However, the peak warmth of Holocene summers, depending on the model or proxy used, is often proposed to begin *ca*. 9,000–7,000 cal BP and last until *ca*. 5,000 cal BP, and is attributed to the dynamics of the Laurentide and Greenland ice sheets^1^,^19^,^49^. In contrast, macrofossil data from European high latitudes suggest an earlier start of the warming (Fig. 2) and indicate that the modern *T*_jul_ often exceeded between 11,500 and 8,500 cal BP^22^,^50^,^51^,^52^, earlier than in the pollen-based reconstructions (Fig. 2 and Supplementary Fig. 2).

Independent evidence supports the concept of a warm early Holocene in northernmost Europe. This includes chironomid-based temperature reconstructions from Kola Peninsula, northeast European Russia and northern Fennoscandia^9^,^22^,^41^,^50^,^53^, diatom and geochemical data from northeast European Russia^54^, macrofossil data from Karelia and western Russia^13^,^42^, and sea-surface temperature reconstructions from the northern North Atlantic^52^,^55^. It is important to verify the early Holocene warming and thermal maximum inferred from aquatic macrofossils by a systematic comparison with independent evidence. How general is our inference that the early Holocene was warmer than previously thought? More multi-proxy studies from northern Europe are needed to verify the patterns appearing from the present rather limited data. As with all biological proxies, there are drawbacks associated with macrofossil data, mainly the relatively few macrofossils usually retrieved compared with pollen grains, and the large number of potential false absences due to low macrofossil production, limited dispersal and preservation. However, macrofossils can frequently be identified to species level, which makes it possible to use indicator species, as we have done here. It is also important to critically assess palaeoclimatic reconstructions, being aware of their merits and their drawbacks, so that they can be used to validate model simulations of palaeoclimate, especially in periods of rapid climate change such as during the late-glacial and early-Holocene periods^56^.

In summary, the lake-sediment plant-macrofossil *T*_julM_ from boreo-nemoral to subarctic zones of northern Europe, consistently indicate a warm (>13 °C) early-Holocene exceeding modern *T*_jul_ by about 2 °C in high latitudes until *ca*. 7,500 cal BP. Aquatic and telmatic plants probably responded rapidly to the Holocene warming, closely following the melting ice margin. Even scarce aquatic macrofossil finds testify to the local presence of the plant and can be used as indicator species to reconstruct *T*_jul_. Pollen-based *T*_julP_ are often lower and modern values were exceeded later than in the macrofossil records. The difference between early-Holocene pollen- and macrofossil-based temperature estimates was largest in northernmost Fennoscandia and lasted until 8,500 cal BP when boreal trees spread into the area. Differences in the boreo-nemoral and southern boreal sites were smaller but consistent at the very beginning of the Holocene. This geographical pattern probably reflects the relatively slow northward spread and establishment of trees whose pollen records substantially influence *T*_julP_, thus causing an underestimate in the pollen-based early-Holocene temperatures due to non-climatic factors reflected in the fossil pollen assemblages. Towards the mid-Holocene, the reconstructed *T*_julP_ and *T*_julM_ converge in all study sites.

The discovery of the discrepancy between macrofossil and pollen reconstructions has implications for palaeoclimate history in northern Europe; perhaps, the impacts of high summer insolation during the early Holocene have been underestimated, especially in the Arctic. This result needs validating by more multi-proxy studies involving pollen and macrofossils, and also by other independent proxies capable of providing a summer temperature reconstruction, such as chironomid records. Simulations from palaeoclimate models to reconstruct climate processes over the last glacial termination are frequently validated against pollen-inferred temperatures. If early-Holocene temperatures really were higher than those reconstructed from pollen data, this modifies our understanding of climate evolution from late-glacial to early-Holocene conditions. Palaeoclimate models will have to be reassessed and validated against the new palaeoclimate scenario. If the models can successfully reproduce past climate changes, we can be more certain of future climate predictions made by the models.

## Methods

### Material

The sediment sequences derived from several sites are located in Fennoscandia, the Baltic region and northeastern European Russia (Fig. 1 and Supplementary Table 1), and span the early Holocene period from 11,700 to 7,500 cal BP. We first use the modern Finnish plant species distribution data in combination with meteorological climate normals (Supplementary Fig. 4 and Supplementary Table 2) to identify the species-specific current lowest requirement for mean *T*_jul._ These limiting temperature values are used to reconstruct macrofossil-based mean *T*_jul_. It should be noted that this procedure can only provide a lowest mean *T*_jul_ estimate. Age–depth models based on calibrated ^14^C dates are available for almost every site (Supplementary Methods) and these original models are applied in this study.

### Pollen-based mean *T*_jul_ reconstructions

Pollen-based *T*_jul_ reconstructions are derived using two-component weighted averaging-partial least squares calibration models^57^. Three regional pollen–climate calibration data sets were used (Fig. 1), selected from the surface-sample data set of Salonen *et al*.^58^ and covering different sections of northern Europe. With each fossil data set, the regional calibration data set containing the fossil site was used, to best represent the climatic responses of taxa within the region in question^59^,^60^. The performance of each calibration model was tested by leave-one-out cross-validation, suggesting a root-mean-square error of predictionof 1.13 °C with calibration data set A, 0.83 °C with set B and 0.84 °C with set C. Sample-specific s.e. were estimated for the reconstructed values using bootstrapping^61^ (100 iterations). The time-window-specific *T*_julP_ value for each site is calculated as the median of all reconstructed values from fossil samples dating to the time window in question. Reconstructions were prepared in C2 software^62^ using all terrestrial pollen and spore taxa, and square-root-transformed species data.

### Macrofossil-based *T*_jul_ reconstructions

A unique modern species-specific spatial plant distribution data set (http://www.luomus.fi/kasviatlas)^63^ covers the whole of Finland and long-term meteorological climate normals are readily available^64^ (Supplementary Fig. 4 and Supplementary Table 2). The plant distribution database can be used to correlate modern species distributions with climate variables, as it is based on continuous botanical surveys. The database is frequently updated and contains over 5.8 million observations, the earliest of which go back to the late nineteenth century.

The macrofossil taxa listed below were used to reconstruct temperature. The modern lowest *T*_jul_ limit (°C) is indicated in brackets (see also Supplementary Table 2). References are ref. 63 and ref. 64, if not stated otherwise: *Cristatella mucedo* (bryozoan remain; 10 °C)^65^, *Nuphar* (13.14 °C), *Nymphaea* (13.49 °C), narrow-leaved *Potamogeton* spp. (a combination of species such as *Potamogeton pusillus*, *Potamogeton rutilus* and *Potamogeton friesii*; 13.61 °C), *Potamogeton compressus* (13.85 °C), *Callitriche cophocarpa* (13.65 °C),*Callitriche hermaphroditica* (14 °C), *Ceratophyllum* (14.11 °C), *Elatine hydropiper* (14.24 °C), *Typha latifolia* (15.69 °C), *Glyceria lithuanica* (15.65 °C), *Najas flexilis* (16.75 °C).

When *Potamogeton* species were not originally identified to species level, we assumed that species assemblages were similar to those detected at the other northern study sites (Supplementary Fig. 5) and we assigned a cautious tentative *T*_jul_ lowest inference value of 13.5 °C. One of the detected species in Russia was *Elatine hydropiper*, which, according to den Virtuella floran, does not currently occur in northeastern European Russia at all, as it is restricted to the western and southwestern parts of European Russia, mainly south of 60°N. This distribution map is unlikely to be accurate but justifies the use of the Finnish distributional data, with an option that in more continental Russia the presence of this species might indicate values higher than 13 °C *T*_jul_. The other taxon that should be mentioned in this context is *Typha*. *T. latifolia* has a more northern distribution range than *T. angustifolia*, and because *Typha* seeds cannot be identified to species level we assume that the species in fossil records is *T. latifolia*. In European Russia the northern limit of the continuous presence of *T. latifolia* follows *ca*. 17 °C isotherm, whereas in Finland the restricting *T*_jul_ is 15.69 °C. Here we use the *T*_jul_ value from Finland as for all other species.

Even though the macrofossil-derived reconstructions are not continuous in the same way as pollen reconstructions—they are based on the presence of indicator species and often samples do not contain any remains that may be used to infer temperature—the temperature reconstructions are displayed as interpolated (dashed) lines to facilitate comparison with the pollen–climate reconstructions (Supplementary Fig. 2).

The macrofossil-based *T*_jul_ reconstruction includes the following steps: first, the most indicative aquatic or lake-shore plant species were selected from the original macrofossil assemblages (Supplementary Fig. 3). Next, modern species distribution observations^63^ and a mean *T*_jul_ derived from the same grid cells, based on daily measurements (1970–2000) by the Finnish Meteorological Institute^64^, were used to estimate current species-specific *T*_jul_ limits (Supplementary Fig. 4 and Supplementary Table 2). The species-specific *T*_jul_ limit was estimated as the median of the mean *T*_jul_ values in grid cells containing individual species occurrences along the northernmost modern distribution limit. The *T*_julM_ palaeo-curve (Supplementary Fig. 2) was then created based on the macrofossil assemblages using the lowest requirements for mean *T*_jul_ for the fossil taxa found. For *T*_julM_ estimates for each time window (Fig. 2), we selected that taxon from the assemblages, which has the highest modern mean *T*_jul_ limit in the north. For example, if samples within the 9,500–8,500 cal BP time window contained *Nymphaea*, *C. hermaphroditica* and *Typha* seeds, the 9,500–8,500 cal BP time-window *T*_julM_ was derived as the modern mean *T*_jul_ requirement of *Typha*, which is higher than that of *Nymphaea* and *C. hermaphroditica*.

### Assessment of errors

For pollen-based *T*_jul_ values, the bootstrap-estimated s.e. vary from sample to sample. The core-specific mean s.e. are 0.85–0.95 °C for the Fennoscandian and Baltic sites, while for the Russian sites the mean s.e. are somewhat higher at 1.05–1.15 °C, probably because the Russian calibration data set is smaller and the species-response estimates are thus less certain. In practice, pollen-based reconstructions tend to have a significant amount of sample-to-sample noise (see for example, Supplementary Fig. 1a), for example, due to the random variability in (a) the selection of pollen grains for the sample count, (b) taphonomic processes and (c) non-climatic factors affecting pollen production. For this reason, the climatic value of a single pollen assemblage can be dubious; hence, pollen-based stratigraphic reconstructions are commonly smoothed using locally weighted regression, as is done here (Supplementary Fig. 2). To derive the time-window-specific *T*_julP_ value, we used the median of the sample-specific *T*_jul_ values falling within the time window, to represent a typical pollen-based *T*_jul_ value within the time window. In doing this, we assume that the sample-to-sample variation within the time window is largely noise and not real climatic variability, which is probably justified due to the relative shortness of the time windows.

Sample-specific error estimations as with pollen are not possible with the macrofossil indicator species method. However, we consider that the method used here most likely provides an underestimate rather than an overestimate of the reconstructed species-specific mean *T*_jul_. The reconstructed species-specific mean *T*_jul_ is based on the median of modern mean *T*_jul_ observations at grid cells containing individual occurrences at the modern northern species limit. The median value incorporates *T*_jul_ values of all individual outlying occurrences, including those that may be situated in unusually favourable microhabitats with an ideal *T*_jul_ microclimate and/or an ideal combination of secondary ecologically significant environmental factors. Thus, we consider it improbable that a generally applicable species-specific *T*_jul_ requirement would be lower than the value derived here and, by extension, we consider the derived *T*_julM_ values to be conservative (that is, low). A measure of the largest conceivable *T*_julM_ overestimation can be derived from the differences between the ‘median observed' *T*_jul_ and the ‘lowest observed' *T*_jul_ at the northern distribution limit for each species. These median-to-lowest differences vary from 0.02 to 1.24 °C, with a mean of 0.50 °C (Supplementary Table 2). Thus, if the *T*_julM_ estimates were based on the absolute lowest *T*_jul_ at which the species has been observed—which would almost certainly be an underestimate for the reasons noted above—the *T*_julM_–*T*_julP_ anomalies would not fall by more than a few 0.1 °C, and especially the large early-Holocene anomalies of >2 °C would remain much higher than the estimated s.e. of the pollen-based reconstruction.

In most cases, the proxies were analysed from the same sediment sections, that is, both proxy data are based on same age-depth models, and uncertainties related to the original chronologies or age-depth model establishment should thus be minor.

### Interpretation of reconstructions

It should be noted that the *T*_julM_ values derived here represent lowest mean *T*_jul_ estimates only, and that possible local presence of any taxa with higher *T*_jul_ requirements cannot be excluded based on the absence of macrofossils, due to the possibility of so-called ‘false absences' in the plant macrofossil record^66^. By comparison, false absences are not likely with pollen data due to the much greater production and even dispersal of pollen grains compared with macroscopic plant remains, and with *T*_julP_ taphonomic processes alone are no more likely to cause a negative bias compared with a positive one. Owing to the nature of *T*_julM_ as a lowest estimate for mean *T*_jul_, negative *T*_julM_–*T*_julP_ anomalies are difficult to interpret, as it is impossible to verify whether the anomaly is due to a false absence of macrofossils requiring greater warmth or some other underlying bias in either the macrofossil or pollen-based technique. By comparison, positive *T*_julM_–*T*_julP_ anomalies can be more confidently taken to suggest a negative bias in the *T*_julP_ values; especially as for methodological reasons (see previous subsection) the *T*_julM_ values are not likely to be substantially overestimated. In our analysis, we thus pay particular attention to the spatio-temporal incidence of situations where the *T*_julM_ estimate exceeds the *T*_julP_ value (positive *T*_julM_–*T*_julP_ anomaly) considerably (by>2 °C), thus strongly suggesting an unaccounted for source of negative bias in the pollen-based reconstruction.

## Author contributions

M.V. had the main responsibility for the design and writing of the manuscript together with H.H.B., J.S.S. and M.H. J.S.S. performed all the quantitative pollen temperature reconstructions. All co-authors participated, commented and provided contributions relevant to their own data.

## Additional information

**How to cite this article:** Väliranta, M. *et al*. Plant macrofossil evidence for an early onset of the Holocene summer thermal maximum in northernmost Europe. *Nat. Commun.* 6:6809 doi: 10.1038/ncomms7809 (2015).

## Supplementary Material

Supplementary Information Supplementary Figures 1-5, Supplementary Tables 1-3, Supplementary Methods and Supplementary References
