# Supplementary material for: Plant macrofossil evidence for an early onset of the Holocene summer thermal maximum in northernmost Europe
Source: Nat Commun. 2015 Apr 10;6:6809. doi: 10.1038/ncomms7809 (PMC4403309; doi:10.1038/ncomms7809)
Supplement: Supplementary Information — Supplementary Figures 1-5, Supplementary Tables 1-3, Supplementary Methods and Supplementary References [file ncomms7809-s1.pdf]

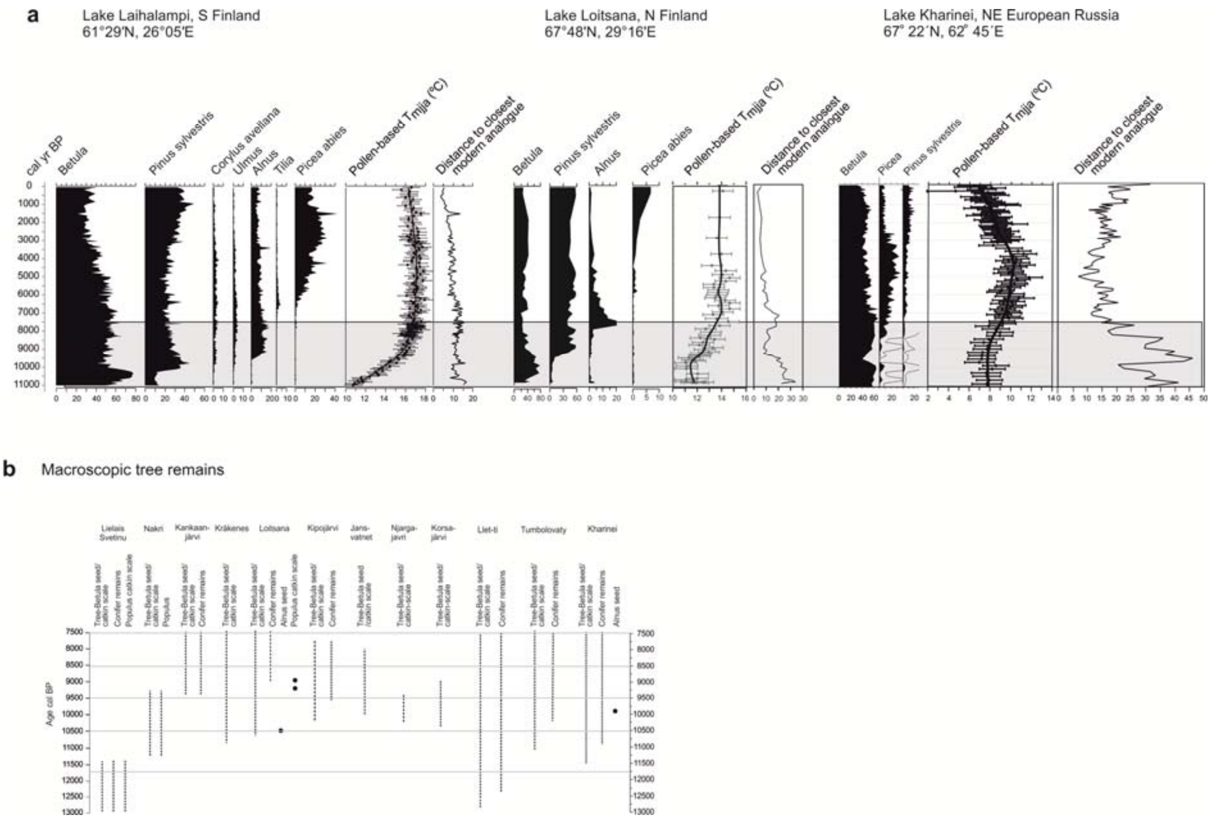

Supplementary Figure 1. Pollen (three examples) and macrofossil data of tree species from the study sites. (**a**) Three example diagrams demonstrating how the rise in pollen-based reconstructed July temperatures follows the proportional increase of certain tree pollen: temperate tree species in the southern boreal zone, pine in northern Fennoscandia, and spruce in European Russia. Fossil assemblage distances from closest modern analogue are also shown, highlighting the longer distances in the early Holocene. The error bars for pollen-based temperature values represent sample-specific bootstrap-estimated standard errors[1].

(**b**) Macroscopic tree remain finds from the studied sediment sections. We do not know if the tree-type birch seeds originated from mountain birch (*Betula pubescens* ssp. *czerepanovii*), pubescent birch (*Betula pubescens*) or silver birch (*Betula pendula*), but the lowest possible T_{jul} which is the delimiting temperature for mountain birch is 10°C. This temperature can also

be applied to *Populus* and *Alnus* tree lines. In north-eastern European Russia the current regional T_{jul} range limit for spruce, which forms the conifer tree line, is 13.5°C[2]. In Fennoscandia the tree-line conifer is *Pinus sylvestris* and this tree-limit follows the 12.2˚C isotherm[3].

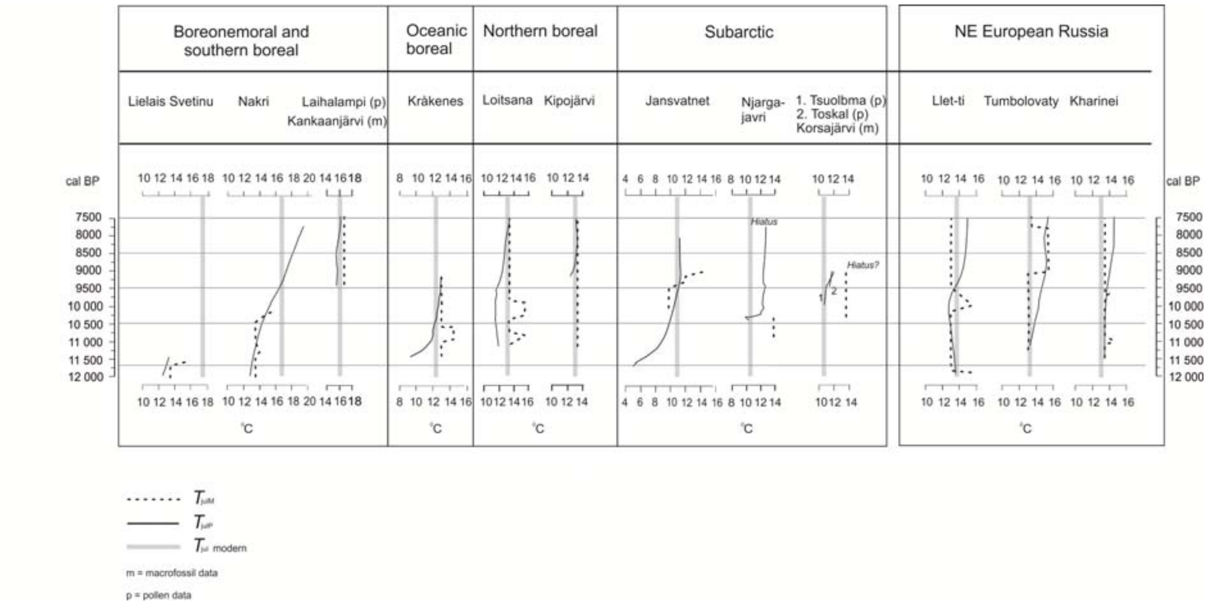

Supplementary Figure 2. July temperature reconstructions for each study site. The dashed line is the macrofossil-based temperature reconstruction (T_{julM}) and the solid line is the pollen-based temperature reconstruction (T_{julP}). The grey bars indicate the modern July temperature. Llet-Ti pollen and macrofossils originate from different but adjacent sediment cores.

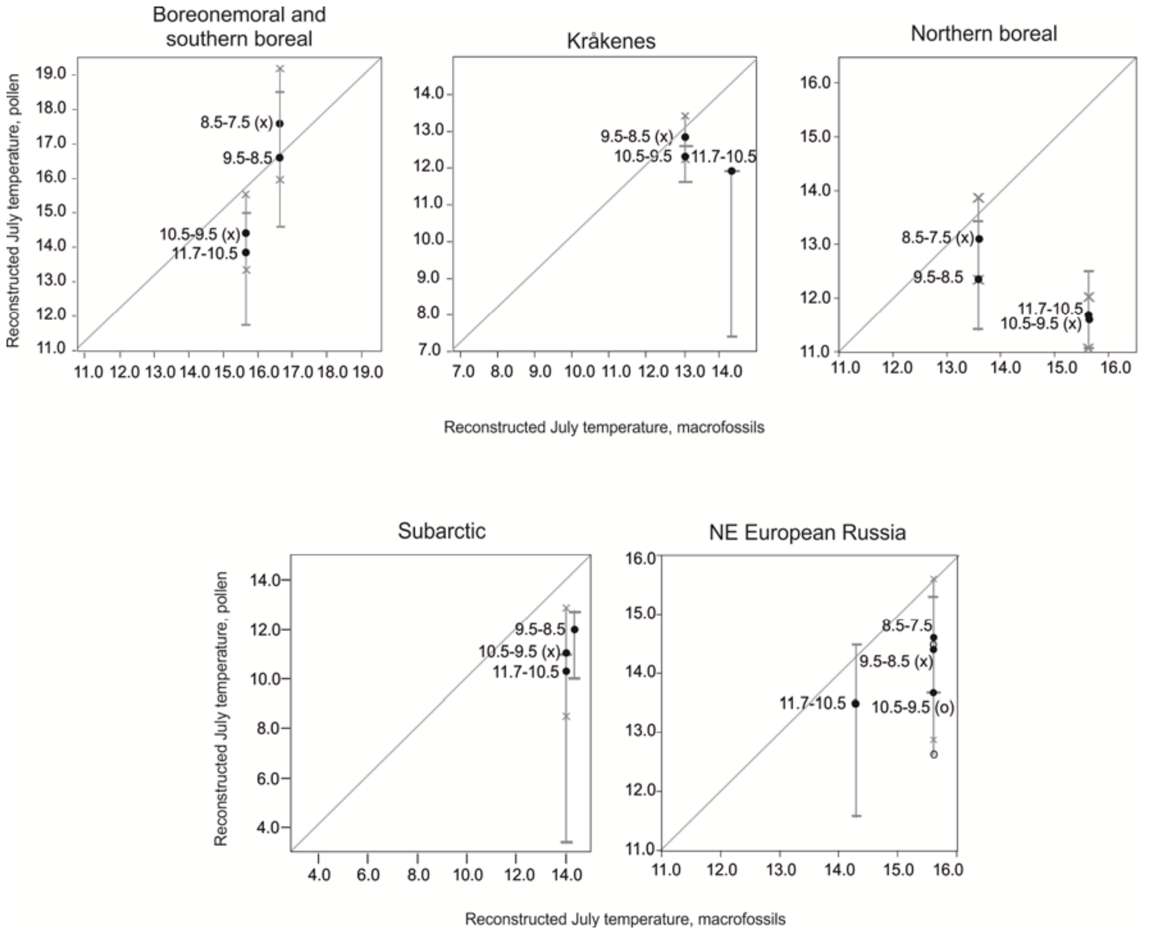

Supplementary Figure 3. A scatter plot presentation of regional-scale early-Holocene temperature development. The study sites were grouped into regional clusters and the focus time period was divided into four time windows. Each black dot indicates, for one time window, the mean of the site-specific medians for pollen-based July temperature (y axis) and the highest estimate for minimum macrofossil-based July temperature from all sites in the site cluster (x axis). Minimum and maximum pollen-based temperature estimates from the site cluster per time window are shown as crosses, circles or horizontal bars. Macrofossil data cannot provide such ranges. For more detailed description of macrofossil-based reconstructions see Methods. Note that the "Subarctic" panel uses a different temperature scale compared with the other panels.

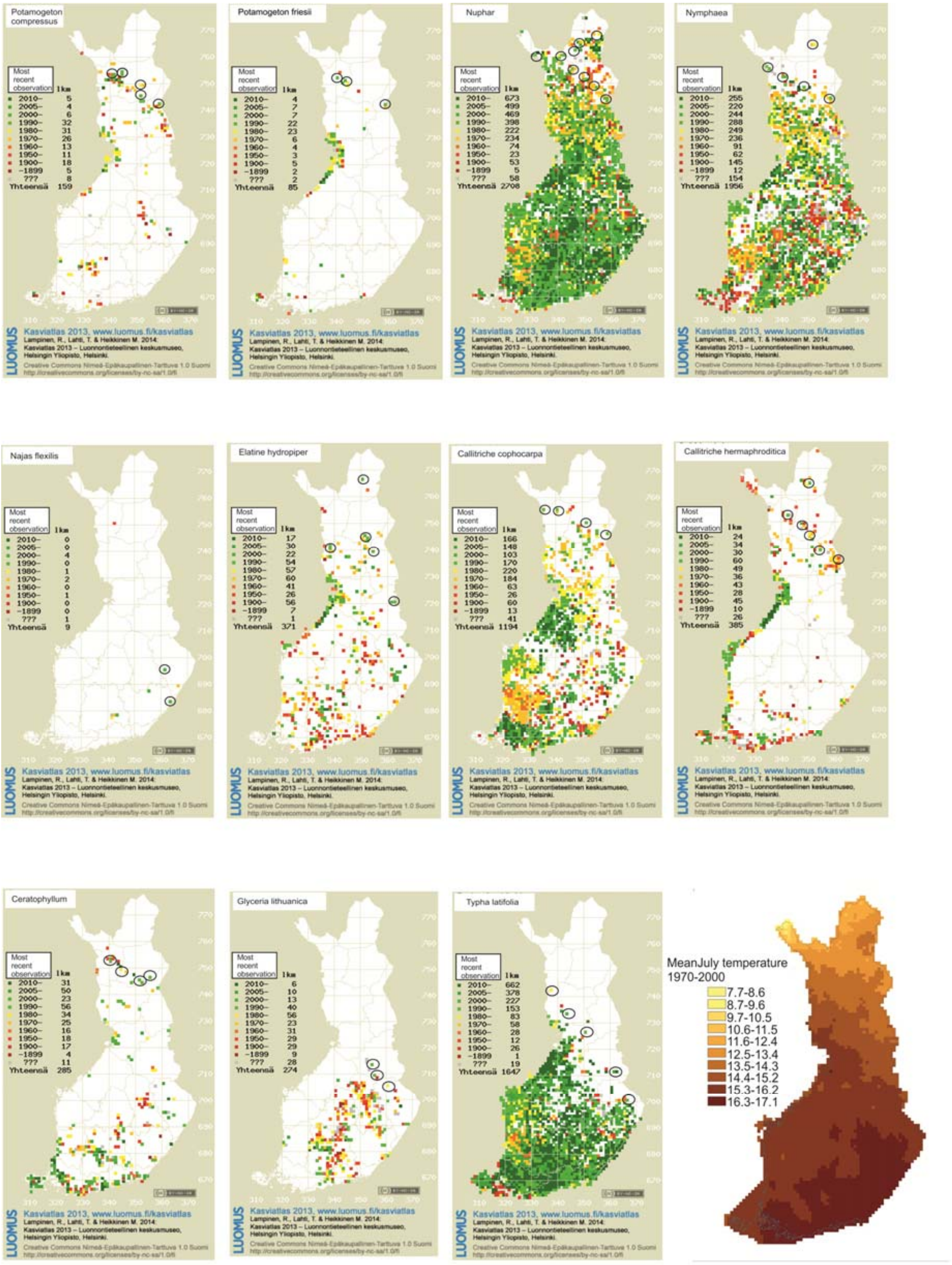

Supplementary Figure 4. Thematic presentation of the methodology used to derive macrofossil-based lowest mean July temperature estimates. The species-specific lowest mean

July temperature estimates were achieved by selecting a set of species observations that are located at the northernmost border of the current distribution range (circles). We only accounted for observations made during 1970-2000 (light green and yellow symbols), which correspond to the climate normals period of the meteorological data. In the data base[4] all plant observations have x and y coordinates, thus all observations can be linked to the meteorological data derived from the same grid cells[5]. These combined data were used to estimate the lowest mean July temperature range where the species currently occurs and subsequently to reconstruct past July temperatures.

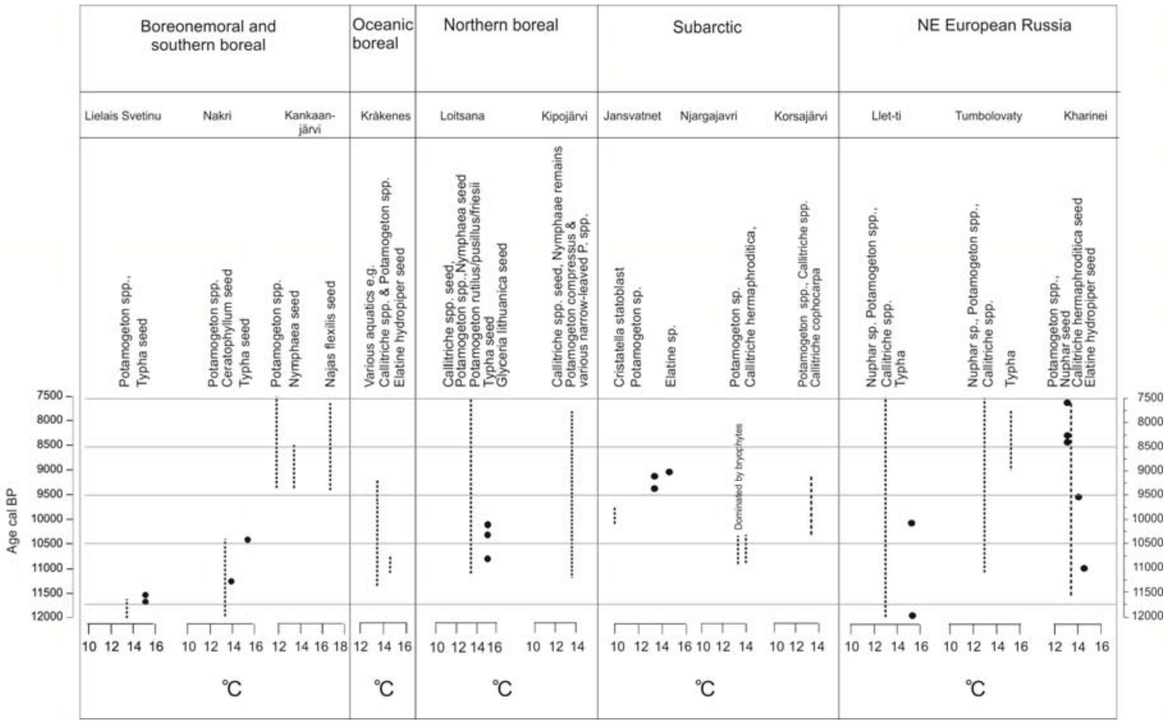

Supplementary Figure 5. Site-specific information of macrofossil taxa that were used to reconstruct the lowest possible July temperatures (T_{julM}). In Jansvatnet aquatic or helophyte species with a clear temperature indication value were not present before 9500 cal yr BP so *Cristatella* statoblasts were used. Narrow-leaved *Potamogeton* spp. includes species such as *P. pusilus*, *P. rutilus,* and *P. friesii*. A black dot indicates that the species was present in only

one sample, whereas a more continuous presence in several consecutive samples is indicated by a dashed line.

Supplementary Table 1. Study sites and information on previous publications. Site numbers refer to the locations in Fig. 1.

Site	Size, depth, modern T_{jul}	Macrofossil data	Pollen data	Macrofossil-based temperature reconstruction	Pollen-based temperature reconstruction
1. L. Lielais Svētinu, E Latvia 56°45′N, 27°08′E	19 ha, 4.9 m, 17.7°C	Veski et al. (2012)[6]	Veski et al. (2012)[6]	This study	This study
2. L. Nakri, S Estonia 57°53′N, 26°16′E	1 ha, 3.2 m, 17.0°C	Amon et al. (2012)[7]	Amon et al. (2012)[7]	This study	This study
3. L. Kankaanjärvi, S Finland 60°58′N, 22°38′E	0.55 ha, 7 m, 16°C	Lahtinen (2010) (unpublished)	Not available	This study	Not available
4. L. Laihalampi, S Finland 61°29′N, 26°05′E	25.5 ha, 1.2 m, 16°C	Not availabe	Heikkilä & Seppä (2003)[8]	This study	Heikkilä & Seppä (2003)
5. Kråkenes Lake, W Norway 62°02′N, 5°00′E	2.3 ha, 13 m, 12.6°C	Birks (2000)[9]; Birks & Birks (2013)[10]	Birks & Birks (2013)[10]	Birks & Ammann (2000)[11]	Birks *et al.* (2000)[12]; Bjune *et al.* (2010)[13]
6. L. Loitsana, N Finland 67°48′N, 29°16′E	9 ha, 2 m, 13˚C	Shala (2014)[14]	Salonen et al. (2013)[15]	Shala (2014)[14]	Salonen et al. (2013)[15]
7. L. Kipojärvi, N Finland 69°11′N, 27°17′	11 ha, 1.5 m, 13°C	Väliranta et al. (2011)[16]; Siitonen et al. (2011)[17]	Väliranta et al. (2011)[16]; Siitonen et al. (2011)[17]	Luoto et al. (2014)[18]	This study
8. L. Njarga, N Finland 69°46′ N, 27°20′ E	14 ha, 2 m, 9.5˚C	Väliranta et al. (2005)[19]	Väliranta et al. (2005)[19]	Luoto et al. (2014)[18]	This study
9. L. Korsajärvi, N Finland 68˚49′N, 22˚05′E	20 ha, 1.2 m, 9.5°C	Luoto et al. (2014)[18]	Not available	Luoto et al. (2014)[18]	Not available
10.Jansvatnet, N Norway 70°39′N, 23°40′E	6 ha, 9.2 m, 11°C	Birks et al. (2012)[20]	Birks et al. (2012)[20]	This study	Birks et al. (2012)[20]
11. L. Llet-Ti, NE European Russia 66°31′N, 59°18′E	80 ha, 1.5 m, 13.5°C	Väliranta et al. (2006)[21]	Väliranta et al. (2006)[21]	This study	This study
12. L.Tumbulovaty, NE European Russia 67˚07′N, 59˚33′E	50 ha, 1.6 m, 13.4°C	Kultti et al. (2004)[22]	Kultti et al. (2004)[22]	This study	Salonen et al. (2011)[23]
13. L. Kharinei, NE European Russia 67˚21′N, 62˚44′E	50 ha, 16 m, 13°C	Salonen et al. (2011)[23]; Jones et al. (2011)[24]	Salonen et al. (2011)[23]; Jones et al. (2011)[24]	This study	Salonen et al. (2011)[23]; Jones et al. (2012)[24]

Supplementary Table 2. Sampled modern mean T_{jul} values at the modern species distribution limits.

For each species, we sampled 10x10 km interpolated mean T_{jul} grid (based on[4]) at several grid cells containing species occurrences along the current (observations 1970-2000) distribution boundary[5] (Supplementary Fig. 2). Bold values are the medians of the sampled mean T_{jul} values for each species, and this species-specific T_{jul} limit is used in the macrofossil-based reconstructions. The lowest species-specific mean T_{jul} observation is shown in *Italics*, the highest <u>underlined</u>. *To assign a temperature estimate for the combined narrow-leaved *Potamogeton* group (including for instance *P. rutilus*, *P. pusilus*, and *P. friesii*) we used the species *P. friesii* which currently has the northernmost distribution limit.

Taxon	N:E coordinates	July mean	Taxon	N:E coordinates	July mean
Potamogeton Compressus	7535000:3415000	13.66	Nymphaea	7565000:3345000	13.37
	7495000:3515000	13.85		7525000:3415000	13.61
	7545000:3445000	*13.13*		7645000:3515000	13.49
	7455000:3515000	<u>14.28</u>		7545000:3525000	*13.23*
	7425000:3585000	13.87		7445000:3585000	*13.98*
		**13.85**			**13.49**
Narrow-leaved Potamogeton*	7525000:3415000	13.61	Najas flexilis	6955000:3615000	*16.7*
	7515000:3435000	*13.59*		6835000:3635000	<u>16.8</u>
	7425000:3585000	<u>13.87</u>			**16.75**
		**13.61**	Elatine hydropiper	7415000:3385000	14.75
Nuphar	7685000:3535000	13.01		7445000:3515000	14.24
	7605000:3315000	*12.48*		7395000:3555000	14.15
	7605000:3425000	12.55		7205000:3625000	<u>15</u>
	7625000:3465000	12.72		7665000:3505000	*13*
	7655000:3555000	13.37			**14.24**
	7555000:3455000	13.14	Callitriche cophocarpa	7555000:3355000	*13.5*
	7515000:3515000	13.6		7555000:3405000	13.46

	7485000:3545000	13.84		7505000:3515000	13.79
	7445000:3585000	<u>13.98</u>		7475000:3555000	<u>13.89</u>
		**13.14**			**13.65**
Ceratophyllum	7535000:3405000	*13.63*	Callitriche hermaphroditica	766500:3505000	*13*
	7455000:3515000	<u>14.28</u>		7525000:3425000	13.71
	7465000:3555000	14.11		7495000:3475000	14
	7425000:3585000	13.87		7435000:3515000	14.26
	7455000:3515000	<u>14.28</u>		7395000:3545000	<u>14.54</u>
		**14.11**		7335000:3615000	14.19
Typha	7335000:3435000	*15.08*			**14**
	7265000:3505000	15.39	Glyceria lithuanica	7135000:3545000	15.65
	7115000:3615000	15.69		7095000:3555000	*15.4*
	7005000:3675000	15.79		6905000:3685000	<u>16.11</u>
	6945000:3695000	<u>15.97</u>			**15.65**
		**15.69**			

Supplementary Table 3. Pollen- and macrofossil based T_{jul} reconstructions and T_{julM} - T_{julP} anomalies for each site and each time window.

The time window-specific T_{julP} value was calculated as the median of reconstructed T_{jul} values for the samples falling within the time window. T_{julM} is the highest species-specific minimum requirement for mean T_{jul} among the indicator species found as macrofossils within the time-window.

Site	T_{julP}				T_{julM}				$T_{julM}–T_{julP}$			
	11.7–10.5	*10.5–9.5*	*9.5–8.5*	*8.5–7.5*	*11.7–10.5*	*10.5–9.5*	*9.5–8.5*	*8.5–7.5*	*11.7–10.5*	*10.5–9.5*	*9.5–8.5*	*8.5–7.5*
Jansvatnet	8.97	10.71	11.97	10.86	N/A	10	14.24	N/A	N/A	-0.71	2.27	N/A
Kharinei	13.55	13.22	13.05	14.27	14.24	14	13.3	13.14	0.69	0.78	0.45	-1.13
Kipojärvi	N/A	N/A	12.04	12.70	13.38	13.38	13.38	13.38	N/A	N/A	1.81	1.15
Kråkenes	10.25	12.15	12.41	N/A	14.24	13	13	N/A	3.99	0.85	0.59	N/A
Laihalampi(P)/Kankaanjärvi(M)	11.55	13.68	15.99	16.63	N/A	N/A	16.75	16.75	N/A	N/A	0.76	0.12
Lielais Svetinu	13.40	N/A	N/A	N/A	15.69	N/A	N/A	N/A	2.29	N/A	N/A	N/A
Llet-Ti	12.67	13.98	14.67	14.86	13.14	15.69	13.14	13.14	0.47	1.71	-1.53	-1.72
Loitsana	11.67	11.62	12.35	13.07	15.65	15.69	13.61	13.61	3.98	4.07	1.26	0.54
Nakri	12.78	14.20	16.46	17.93	14	15.69	N/A	N/A	1.33	1.49	N/A	N/A
Njargajavri	N/A	11.95	12.47	12.72	14	14	N/A	N/A	N/A	2.05	N/A	N/A
Tumbulovaty	13.70	14.01	15.45	14.80	13	13	15.69	15.69	-0.7	-1.01	0.24	0.89
Tsuolbma+Toskal(P)/Korsa(M)	11	11	11.5	N/A	N/A	13.65	13.65	N/A	N/A	2.65	2.15	N/A

Supplementary Methods

Chronological information and age-depth models

For this study we used mainly existing and published age-depth models and here we provide only basic information. The more detailed descriptions of the chronological procedures can be found from the original publications, see Supplementary Table 1. Only the Llet-Ti and Njargajavri age-depth models were newly created.

L. Lielais Svētinu: The chronology of Lake Lielais Svētinu is based on 12 samples of mixed terrestrial plant material[6]. Samples were dated by the accelerator mass spectrometry (AMS) method and the BP dates were calibrated, and an age–depth model was built with an OxCal 4.1 depositional model, including visible sedimentary boundaries[25,26].

L. Nakri: The chronology of Lake Nakri is based on 9 samples of mixed terrestrial plant material[7]. Samples were dated by the AMS method and the BP dates were calibrated, and an age–depth model was built with an OxCal 4.1 depositional model, including visible sedimentary boundaries[25,26].

L. Kankaanjärvi: The Kankaanjärvi chronology (unpublished) is based on six samples of terrestrial plant material. Samples were dated by the AMS method and BP dates were calibrated using CALIB 5.0 program[27] and the age-depth model was created by applying five-order polynomial fitting.

L. Laihalampi: Laihalampi chronology is based on five bulk sediment samples[8]. Samples were dated by conventional ^{14}C method and the BP dates were calibrated using CALIB 4.2 program[27,28]. The age-depth model was created using a non-parametric weighted regression within the framework of generalized additive models (GAM).

Kråkenes Lake: The chronology is based on 96 AMS dates on samples of terrestrial plant material. The dates were calibrated using IntCal09 and the age-depth model[29] was created by the OxCal program V 4.1[30].

L. Loitsana: Loitsana chronology is based on ten samples of terrestrial plant material[14]. The samples were dated by AMS method. The BP dates were calibrated and the age-depth model was calculated in R (R Core Team 2011) using the CLAM package[31] and the IntCal09 calibration curve[32,33].

L. Kipojärvi: The Kipojärvi chronology is based on eight samples of terrestrial plant material[16,17]. The samples were dated by AMS method. The BP ages were calibrated using CALIB 601 and the IntCal09 calibration curve[27,32]. Age-depth model was created by fitting a second-order polynomial curve to the calibrated dates.

L. Njargajavri: Altogether eight plant material samples were dated[19]. Six dated samples were composed of terrestrial plants while two contained of aquatic mosses. The samples were dated by AMS method. The BP ages were calibrated using the program CALIB 4.4[27]. The chronological data supported by pollen stratigraphical comparison suggested a hiatus and only the section below the hiatus was included to this study. A cubic smooth spline age–depth model was fitted to the ^{14}C and pollen dates using the R function[34].

L. Korsajärvi: Altogether nine samples containing terrestrial plant material were dated (unpublished). The samples were dated by AMS method. The BP ages were calibrated with the software CALIB 601 using the IntCal09 calibration curve[27,33]. The chronological data suggested a hiatus and only the section below the hiatus was included to this study. A linear interpolation between the dated levels was used to create the age-depth model[18].

Jansvatnet: the chronology is based on 6 AMS radiocarbon date on terrestrial plant material and calibrated using IntCal09. The date of deglaciation in northern Norway and the GICC05

date for the start of the Holocene were also used and the age-depth model was drawn by linear interpolation[20].

L. Llet-ti: Macrofossil core[21]: the chronology is based on five bulk sediment samples (conventional method) and three terrestrial plant material samples (AMS method). The dates were calibrated using the program CALIB 4.4[27] and linear interpolation was applied to create the age-depth model. Pollen core: the chronology (unpublished) is based on seven terrestrial plant remain samples dated by AMS ^{14}C. The dates were calibrated in Oxcal 4.0 using the IntCal09 calibration curve[35], and a cubic smooth spline age–depth model was fitted to the dates using the R function[34].

L. Tumbulovaty: The chronology is based on three bulk sediment samples (conventional method) and two terrestrial plant remain samples (AMS method)[22]. Linear interpolation between calibrated radiocarbon, version CALIB 4.4, ages[27] was used to create the age-depth model.

L. Kharinei: The chronology of the Lake Kharinei is based on six samples of mixed terrestrial plant material[23]. Samples were dated by the AMS method and dates were calibrated with OxCal 4.0[35]. A cubic smooth spline age-depth model was fitted to the dates using the R function[34].

Supplementary References

1. Birks, H. J. B., Line, J. M., Juggins, S., Stevenson, A. C. & ter Braak, C. J. F. Diatoms and pH reconstruction. *Phil. Trans. r. Soc., Lond. B.* **327**, 263–278 (1990).
2. Virtanen, T. *et al.* Modelling the location of the forest line in northeast European Russia with remotely sensed vegetation and GIS-based climate and terrain data. *Arct. Antarct. Alp. Res.* **36**, 314–322 (2004).
3. Kultti, S., Mikkola, K., Virtanen, T., Timonen, M. & Eronen, M. Past changes in the Scots pine forest line and climate in Finnish Lapland: a study based on megafossils, lake sediments, and GIS-based vegetation and climate data. *Holocene* **16**, 381-391 (2006).
4. Venäläinen, A., Tuomenvirta, H., Pirinen, P. & Drebs, A. *A basic climate data set 1961-2000 - description and illustrations*. Finnish Meteorological Institute, Reports 5. (2005).
5. Lampinen, R. & Lahti, T. 2013: *Kasviatlas*. Helsingin Yliopisto, Luonnontieteellinen keskusmuseo, Helsinki. (2012).
6. Veski, S., Amon, L., Heinsalu, A., Reitalu, T., Saarse, L., Stivrins, N. & Vassiljev, J. Lateglacial vegetation dynamics in the eastern Baltic region between 14,500 and 11,400 cal yr BP: A complete record since the Bølling (GI-1e) to the Holocene. *Quat. Sci. Rev.* **40**, 39-53 (2012).
7. Amon, L., Veski, S., Heinsalu, A. & Saarse, L. Timing of Lateglacial vegetation dynamics and respective palaeoenvironmental conditions in southern Estonia: evidence from the sediment record of Lake Nakri. *J. Quat. Sci.* **27**, 169-180 (2012).
8. Heikkilä, M. & Seppä, H. A 11,000 yr palaeotemperature reconstruction from the southern boreal zone in Finland. *Quat. Sci. Rev.* **22**, 541–554 (2003).

9. Birks, H. H. Aquatic macrophyte vegetation development in Kråkenes Lake, western Norway, during the late-glacial and early-Holocene. *J. Paleolim.* **23**, 7-19 (2000).

10. Birks, H. H. & Birks, H. J. B. Vegetation responses to late-glacial climate changes inwestern Norway. Preslia **85,** 215–237 (2013)

11. Birks, H. H. & Ammann, B. Two terrestrial records of rapid climatic change during the glacial–Holocene transition (14,000– 9,000 calendar years B.P.) from Europe. *P. Natl. Acad. Sci. USA.* **97**, 1390-1394 (2000).

12. Birks, H. H. *et al.* The development of the aquatic ecosystem at Kråkenes Lake, western Norway, during the late-glacial and early-Holocene – a synthesis. *J. Paleolim* **23,** 91–114 (2000).

13. Bjune, A. E., Birks, H. J. B., Peglar, S. M., Odland, A. Developing a modern pollen-climate calibration data set for Norway. *Boreas* **39**, 674-688 (2010).

14. Shala, S. *Palaeoenvironmental changes in the northern boreal zone of Finland: local versus regional drivers*. Dissertation from the Department of Physical Geography and Quaternary Geology No 40. Stockholm University. ISBN 978-91-7447-838-9. (2014).

15. Salonen, J. S., Helmens, K. F., Seppä, H. & Birks, H. J. B. Pollen-based palaeoclimate reconstructions over long glacial–interglacial timescales: Methodological tests based on the Holocene and MIS 5d–c deposits of Sokli, northern Finland. *J. Quat. Sci.* **28,** 271–282 (2013).

16. Väliranta, M. *et al.* Holocene aquatic ecosystem change in the boreal vegetation zone of northern Finland. *J. Paleolim.* **45**, 339-352 (2011).

17. Siitonen, S., Väliranta, M., Weckström, J., Juutinen, S. & Korhola, A. Comparison of Cladocera-based water-depth reconstruction against other types of proxy data in Finnish Lapland. *Hydrobiologia* **676**, 155–172 (2011).

18. Luoto, T., Kaukolehto, M., Weckström, J., Korhola, A. & Väliranta, M. New evidence of warm early-Holocene summers in subarctic Finland based on an enhanced regional chironomid-based temperature calibration model. *Quat. Res.* **81**, 50-62 (2014).

19. Väliranta, M., Kultti, S., Nyman, M. & Sarmaja-Korjonen, K. Holocene development of aquatic vegetation in a shallow Lake Njargajavri, Finnish Lapland with evidence of water level fluctuations and drying. *J. Paleolim.* **34**, 203-215 (2005).

20. Birks, H. H. *et al.* From cold to cool in northernmost Norway: Lateglacial and early Holocene multi-proxy environmental and climate reconstructions from Jansvatnet, Hammerfest. *Quat. Sci. Rev.* **33**, 100-120 (2012).

21. Väliranta, M., Kultti, S. & Seppä, H. Vegetation dynamics during the Younger Dryas - Holocene transition in the extreme northern taiga zone, north-eastern European Russia, *Boreas* **35**, 202-212 (2006).

22. Kultti S., Oksanen, P. & Väliranta, M. Holocene tree line, permafrost, and climate dynamics in the Nenets Region, East European arctic. *Can. J. Earth Sci.* **41**, 1141-1158 (2004).

23. Salonen, J. S. *et al.* Holocene and lateglacial temperature changes and associated treeline dynamics in NE European Russia. *Quat. Res.* **75**, 501-511 (2011).

24. Jones, V. J. *et al.* The influence of Holocene treeline advance and retreat on an arctic lake ecosystem; a multi-proxy study from Kharinei Lake, North Eastern European Russia. *J. Paleolim.* **46**, 123-137 (2011).

25. Reimer, P. J., Baillie, M. G. L., Bard, E. *et al.* IntCal04 terrestrial radiocarbon age calibration, 0–26 cal kyr BP. *Radiocarbon* **46**, 1029–1058 (2004).

26. Bronk Ramsey, C. Deposition models for chronological records. *Quat. Sci. Rev.* **27**, 42–60 (2008).

27. Stuiver, M. & Reimer, P.J. Extended 14C data base and revised CALIB 3.2 14C age calibration program. *Radiocarbon* **35**, 215–230 (1993).

28. Stuiver, M. *et al.* 1998 INTCAL98 radiocarbon age calibration, 24,000–0 cal BP. *Radiocarbon* **40**, 1041–1083 (1998).

29. Lohne, Ø., Mangerud, J., Birks, H. H. Precise ^{14}C ages of the Vedde and Saksunarvatn ashes and the Younger Dryas boundaries from western Norway and their comparison with the Greenland Ice Core (GICC05) chronology. *J. Quat. Sci.* **28**, 490-500 (2013).

30. Bronk Ramsey C. OxCal Program, V 4.1. c14.arch.ox.ac.uk/embed.php?File¼oxcal.html. [1 October 2012]. (2010).

31. Blaauw, M. Methods and code for 'classical' age-modelling of radiocarbon sequences. *Quat. Geochronol*. **5**, 512-518 (2010).

32. Reimer, P.J. *et al.* IntCal09 and Marine09 radiocarbon age calibration curves, 0–50, 000 years cal BP. *Radiocarbon* **51**, 1111–1150 (2009).

33. Bronk Ramsey, C. Bayesian analysis of radiocarbon dates. *Radiocarbon* **51**, 337–360 (2009).

34. Heegaard, E., Birks, H. J. B. & Telford, R. J. Relationships between calibrated ages and depth in stratigraphical sequences: an estimation procedure by mixed-effect regression. *Holocene* **15**, 612–618 (2005).

35. Bronk Ramsey, C. Radiocarbon calibration and analysis of stratigraphy: the OxCal program. *Radiocarbon* **37**, 425–430 (1995).
